# A novel equivalence probability weighted power prior for using historical control data in an adaptive clinical trial design: A comparison to standard methods

**DOI:** 10.1002/pst.2088

**Published:** 2021-01-20

**Authors:** Maxine Bennett, Simon White, Nicky Best, Adrian Mander

**Affiliations:** ^1^ MRC Biostatistics Unit University of Cambridge Cambridge UK; ^2^ Department of Psychiatry University of Cambridge Cambridge UK; ^3^ GlaxoSmithKline Brentford UK; ^4^ Centre for Trials Research Cardiff University Cardiff UK

**Keywords:** adaptive design, Bayesian, borrowing, historical data, priors

## Abstract

A standard two‐arm randomised controlled trial usually compares an intervention to a control treatment with equal numbers of patients randomised to each treatment arm and only data from within the current trial are used to assess the treatment effect. Historical data are used when designing new trials and have recently been considered for use in the analysis when the required number of patients under a standard trial design cannot be achieved. Incorporating historical control data could lead to more efficient trials, reducing the number of controls required in the current study when the historical and current control data agree. However, when the data are inconsistent, there is potential for biased treatment effect estimates, inflated type I error and reduced power. We introduce two novel approaches for binary data which discount historical data based on the agreement with the current trial controls, an equivalence approach and an approach based on tail area probabilities. An adaptive design is used where the allocation ratio is adapted at the interim analysis, randomising fewer patients to control when there is agreement. The historical data are down‐weighted in the analysis using the power prior approach with a fixed power. We compare operating characteristics of the proposed design to historical data methods in the literature: the modified power prior; commensurate prior; and robust mixture prior. The equivalence probability weight approach is intuitive and the operating characteristics can be calculated exactly. Furthermore, the equivalence bounds can be chosen to control the maximum possible inflation in type I error.

## 1 INTRODUCTION

The commonly used two‐arm parallel group randomised controlled trial compares an intervention to a control treatment. Patients are randomised equally to each treatment and the analysis is only based on patients within the current trial. Often, previous trials have included the control treatment and data from these trials could be utilised in the design and analysis of future trials, in particular when the required sample size for the future trial is infeasible to recruit. Incorporating historical control data into the current study analysis could potentially lead to more efficient trials.

In a design where the historical data are used as additional information to supplement the information in the current trial, there is the potential for an increase in power and an increase in the precision of the parameter estimates. Where historical data are used to replace current control data, there is the potential to reduce the sample size and the duration of the current study when the historical outcomes are consistent with those in the current control population. However, when these are inconsistent, there is the potential for biased treatment effect estimates, inflated type I error and reduced power.

An early seminal paper by Pocock^1^ discussed the use of historical controls in the design and analysis of a current trial. This paper posed five important questions: what is relevant historical data; how many additional patients does the historical data provide; how do we assess agreement between historical and current data; what sample size is required for the new study; and how can historical data be incorporated into the analysis.

This paper is structured to address these five questions for binary outcome data. The first question was addressed in the original paper where Pocock^1^ defined six acceptability criteria for using historical data in both the design and analysis of a new study. Throughout this paper we assume that there is one relevant historical study available (although the approaches discussed in this paper can, in principle, be extended to multiple historical studies). The historical study is chosen taking into account the six acceptability criteria defined by Pocock.^1^ Since we assume that there is only one relevant historical study available, the maximum number of additional patients that the historical study may provide is the sample size of the historical study. Depending on the agreement between the historical and current control data, the historical data may be down‐weighted and therefore the additional number of patients that the historical data provides will be reduced. The main focus of this paper is to address the remaining three questions: how to assess agreement between historical and current control data; how to design the current trial given the historical data available; and how to incorporate the historical data into the analysis of a new study.

Viele et al.^2^ provide a review of both frequentist and Bayesian methods proposed in the literature for incorporating historical data into a current study. In this paper we focus on three Bayesian methods from the literature: the power prior and modified power prior^3^, ^4^, ^5^; commensurate prior^6^, ^7^, ^8^; and robust mixture prior.^9^, ^10^ These approaches all work in a similar way, by down‐weighting the historical data depending on the agreement with the current control data, but differ in how the agreement is determined and the rate at which the historical data are discounted. The power prior, modified power prior, commensurate prior and robust mixture prior all have disadvantages. For the fixed power prior it is difficult to choose a value for the power before observing the current trial data. Where the power value represents the level of agreement between the current and historical study. For the modified power prior and commensurate prior it is not intuitive how to choose the prior on the parameter that governs the historical data borrowing and it is unclear when choosing the prior how much influence this prior will have on how much information is borrowed for different levels of agreement with the current control data. Furthermore, the fully Bayesian version of the modified power, the commensurate prior and robust mixture prior all result in a posterior distribution for the control arm that is not a known distribution. Therefore, a method to estimate the effective sample size of the historical data is required. There are various methods available to estimate the effective same size,^11^, ^12^ however each method can give a different estimate. These disadvantages are further discussed and illustrated throughout this paper. The disadvantages of the current approaches motivates the methods proposed in this paper. Our aim for the methods proposed is to have a simple approach to determining the amount of discounting of the historical data based on the agreement between the current and historical control data which is intuitive and simple to discuss with clinicians and also gives a known distribution for which we can easily calculate the effective historical sample size. These designs need to allow control over the maximum possible inflation in type I error and the reduction in power when there is conflict between the historical and current data.

The disadvantages of the approaches available in the literature and the effect that utilising historical data can have on the operating characteristics of the current study, may explain the limited use of these methods in practice. However, for trials where it is not possible to recruit the required number of patients for a standard trial design, for example in rare diseases or a trial in a paediatric population, utilising historical data may be beneficial to inference about a possible treatment effect.

We propose two Bayesian methods for assessing the agreement between historical and current control data when the outcome data are binary, an equivalence probability weight and a weight based on tail area probabilities. These two approaches determine a weight which is used to down‐weight the historical data. In this paper we use the weight based on the agreement directly to down‐weight the historical data. However, some function of this weight may be used to down‐weight the historical data, for example, if the sample sizes differ considerably between the historical and current data, as to not overwhelm the current control data with historical data. Historical control data can be used as additional information while maintaining the sample size of the current study with the aim of increasing power.^2^ Various adaptive designs have been proposed in the literature for incorporating historical control data.^2^, ^8^, ^10^

Here we use the adaptive design proposed by Schmidli et al.,^10^ where historical controls replace yet to be randomised current controls when there is agreement between the historical and current control data. The rationale for this adaptive design is that if at the interim analysis the historical and current controls are in agreement, fewer current controls will be randomised in the second stage of the trial, therefore reducing the sample size and duration of the current study. However, if differences are observed between the current controls and historical data at the interim, the historical data will be more heavily discounted and more current controls will be randomised, safeguarding the sample size required for the final analysis. We utilise the analysis approach of the power prior with the proposed weights as a fixed power to down‐weight the historical data.^3^ The operating characteristics of these two new weights are compared to the methods proposed in the literature for binary outcome data. The advantages and disadvantages of all the approaches are discussed. Finally, we explore how to choose the equivalence bounds and the initial weights for the robust mixture prior to control the maximum type I error across all possible response probabilities in the control arm of the current trial.

The remainder of the paper is structured as follows: Section 2 introduces the methods, including methods proposed in the literature and two new approaches for assessing agreement, the adaptive design is then described and the analysis approach for each method of assessing agreement; Section 3 illustrates the different methods for assessing agreement and how they weight the historical data using an example dataset. A simulation study of an adaptive design is also presented which compares the operating characteristics of the different historical borrowing approaches; finally, Section 4 provides a discussion around the historical data methods considered in this paper.

## 2 METHODS

### 2.1 Notation

Let *x*_*h*_ and *y*_*h*_ be the number of responses and non‐responses in the historical data, respectively. Let *x*_*c*_ and *y*_*c*_ be the number of responses and non‐responses in the current control group and *x*_*t*_ and *y*_*t*_ be the number of responses and non‐responses in the current experimental treatment group. *n*_*h*_ , *n*_*c*_ and *n*_*t*_ are the historical, current control and experimental treatment group sample sizes, respectively. Let *p*_*c*_ and *p*_*t*_ denote the true underlying response probabilities in the control arm and experimental treatment arm in the current study, respectively. For models where it is assumed that the true underlying response probabilities in the current and historical studies are not the same, let *p*_*h*_ denote the underlying true response probability in the historical controls.

The next two sections describe different approaches for assessing agreement between the historical and current control data. All methods discount the historical data when it disagrees with the current control data.

### 2.2 Assessing agreement – Methods proposed in the literature

#### 2.2.1 Power prior

The power prior discounts the historical data by raising the likelihood of the historical data to a fixed power. The power can be thought of as a value that quantifies the expected agreement between the historical data and the current control data and is typically chosen to lie anywhere from zero to one, where zero represents complete disagreement and one represents complete agreement between the current and historical controls. The power prior is given by Reference 3,

$$\pi \left(p_{c}|x_{h},y_{h}\right)\propto L{\left(p_{c}|x_{h},y_{h}\right)}^{\alpha_{0}}\pi_{0}\left(p_{c}\right),$$

where *π*_0_(*p*_*c*_) is the initial prior for *p*_*c*_ before the historical data are observed and $L{\left(p_{c}|x_{h},y_{h}\right)}^{\alpha_{0}}$ is the likelihood of the historical data raised to a fixed power, *α*_0_ . The choice of value for *α*_0_ is important and can be difficult to elicit, *α*_0_ controls the amount of historical data incorporated into the final analysis of the current study and therefore the choice of *α*_0_ has implications on the operating characteristics of the current study.

For binary data, assuming *π*_0_(*p*_*c*_) is a Beta(*c*, *d*) distribution, *α*_0_ is a fixed value and there is only one historical study, then,

$$\pi \left(p_{c}|x_{h},y_{h},c,d\right)\propto p_{c}^{\alpha_{0}x_{h}+c-1}{\left(1-p_{c}\right)}^{\alpha_{0}y_{h}+d-1},$$

which gives a Beta( *α*_0_ *x*_*h*_ + *c* , *α*_0_ *y*_*h*_ + *d* ) prior distribution for *p*_*c*_ . Since for a Beta(*a*, *b*) distribution the parameter *a* is interpreted as the number of responses the parameter *b* and non‐responses, the effective sample size of this prior for *p*_*c*_ is *n*_*h*_ *α*_0_ + *c* + *d* .

Alternatively, *α*_0_ can be treated as a random variable, given a prior distribution and estimated from the data. A scaling constant must be added to the power prior formula when *α*_0_ is treated as a parameter, this is illustrated in Section 2.2.2 and the limitations of allowing *α*_0_ to be a random variable are discussed.

#### 2.2.2 Modified power prior

The modified power prior places a prior on the power *α*_0_ with the aim of learning about the agreement between the historical and current control data from the data itself. This prior contains a normalising constant that is required for correct inference when a prior is placed on *α*_0_ _._ ^4^, ^5^ The modified power prior assumes that the historical data and current control data are estimating the same underlying parameter of interest ( *p*_*h*_ = *p*_*c*_ ). For binary outcome data, the modified power prior has the general form,^4^, ^13^

$$\pi \left(p_{c},\alpha_{0}|x_{h},y_{h}\right)\propto \frac{L{\left(p_{c}|x_{h},y_{h}\right)}^{\alpha_{0}}\pi_{0}\left(p_{c}\right)}{\int L{\left(p_{c}|x_{h},y_{h}\right)}^{\alpha_{0}}\pi_{0}\left(p_{c}\right){\mathrm{dp}}_{c}}\pi \left(\alpha_{0}\right),$$

where *π*_0_(*p*_*c*_) is the initial prior for *p*_*c*_ before the historical data are observed, $L{\left(p_{c}|x_{h},y_{h}\right)}^{\alpha_{0}}$ is the likelihood of the historical data raised to a power *α*_0_ , with prior *π*(*α*_0_). *π*(*α*_0_) is assumed to be a Beta( *a*, *b* ) distribution. A Beta distribution is an intuitive choice for *π*(*α*_0_) since it lies between zero and one and covers a wide variety of shapes depending on the parameter values chosen. In the historical data setting, it is unlikely we would want to give more weight to the historical data than the current control data, therefore a Beta prior that does not allow a weight above one is suitable.

For binary data and only one historical study, the likelihood of the historical data follows a binomial distribution, a Beta(a,b) prior is assumed for the power and an initial Beta(c,d) prior is assumed for *p*_*c*_ . The marginal posterior distribution for the power is given by Reference 5,

$$\pi \left(\alpha_{0}|x_{h},y_{h},x_{c},y_{c}\right)\propto \frac{\Gamma \left(\alpha_{0}n_{h}+c+d\right)\Gamma \left(\alpha_{0}x_{h}+x_{c}+c\right)\Gamma \left(\alpha_{0}y_{h}+y_{c}+d\right)}{\Gamma \left(\alpha_{0}x_{h}+c\right)\Gamma \left(\alpha_{0}y_{h}+d\right)\Gamma \left(\alpha_{0}n_{h}+n_{c}+c+d\right)}\alpha_{0}^{a-1}{\left(1-\alpha_{0}\right)}^{b-1}.$$

There is no closed form for the marginal distribution of the control response probability. Numerical integration could be used for inference to calculate Pr(*p*_*t*_ > *p*_*c*_) in the final analysis. However, when using simulation to determine the operating characteristics of a design, using a fully Bayesian approach can be computationally expensive but takes into account the variability of the power parameter. Here we consider using a summary measure of the marginal distribution of the power as a fixed value. For simplicity and because for an adaptive design we will require a single value for assessing agreement between the historical and current controls to adapt the sample size in the current control group based on the single value of the effective sample size of the historical data, we use the summary measure of the marginal posterior distribution of *α*_0_ as a measure of agreement between the historical and current controls and as a fixed value to down‐weight the historical data, we denote this summary measure ${\tilde{\alpha }}_{0}$.

We consider two quasi‐dichotomous priors for *α*_0_ , a Beta(0.5,0.5) and a Beta(0.3,0.3) and also a flat Beta(1,1) prior, taking the mean of the marginal posterior distribution of *α*_0_ as a fixed value to down‐weight the historical data. Quasi‐dichotomous priors have most of their probability mass at the tails of the distribution. Therefore a Beta(a,a), where *a* → 0, prior on *α*_0_ will push the power towards zero or one, which can improve borrowing of the historical data by utilising more of the historical data when it agrees with the current control data and discounting the historical data more quickly as the difference between the historical data increases compared to a Beta(1,1) prior .^14^ We also consider a Beta(1,1) for *α*_0_ , taking the mode of the posterior distribution to down‐weight the historical data. For binary data this is equivalent to the empirical power prior proposed by Gravestock and Held.^15^ The *EHSS* using the power prior approach is then ${\tilde{\alpha }}_{0}n_{h}$.

#### 2.2.3 Commensurate Prior

The commensurate prior^6^, ^7^, ^8^ assumes different underlying parameters for the response probability in the current and historical controls. The commensurate prior for *p*_*c*_ is a conditional prior distribution, centred at the historical data parameter *p*_*h*_ with an additional parameter *τ* that controls the cross‐study borrowing. The joint distribution of *p*_*c*_ , *p*_*h*_ and *τ* before the current trial is then given by,

$$\pi \left(p_{c},p_{h}|x_{h},y_{h},\tau \right)\propto \pi \left(p_{c}|p_{h},1/\tau \right)\pi_{0}\left(p_{h}\right)L\left(p_{h}|x_{h},y_{h}\right)\pi \left(\tau \right),$$

where a conditional relationship between *p*_*c*_ and *p*_*h*_ is modelled by assuming a conditional prior distribution, *π*(*p*_*c*_| *p*_*h*_, 1/*τ*), that is dependent upon *p*_*h*_ , and a hyperparameter, *τ* , controlling the amount of cross study borrowing. *π*_0_(*p*_*h*_) is the initial prior for *p*_*h*_ before the historical data are observed and *L*(*p*_*h*_| *x*_*h*_, *y*_*h*_) is the likelihood of the historical data. Larger values of *τ* indicate increased commensurability and induce increased borrowing of strength from the historical data.

We assume a binomial distribution for the number of responses in the historical data and current controls and use the logit link function to model the response probabilities ( *logit*(*p*_*h*_) = *μ*_*h*_ , *logit*(*p*_*c*_) = *μ*_*c*_ ). We assume a vague prior for the historical response probability *μ*_*h*_ ∼ *N*(0,100), where *N*(*μ*, *σ* ^2^) denotes a normal distribution with mean *μ* and variance *σ* ^2^ . The current control response probability is centred at the mean of the historical response probability, *μ*_*c*_ ∣ *μ*_*h*_, *τ* ∼ *N*(*μ*_*h*_, 1/*τ*) and we consider two conjugate priors for *τ* , a Gamma(1,0.01) and Gamma(1,0.05), as originally proposed by Hobbs et al.^7^ Note that tau is a parameter for the precision (inverse of the variance).

Posterior samples of the control response probability are used to approximate the posterior distribution using a mixture of Beta distributions via optimisation of the mixture probabilities and Beta parameters as proposed by Schmidli et al.^10^ for the robust meta‐analytic predictive (MAP) prior. A single Beta distribution provides an adequate fit to the data for the example considered here and therefore the effective sample size (*ESS*) of the posterior distribution here is simply the sum of the Beta parameters and the *EHSS* is the *ESS* of the posterior distribution minus the number of controls randomised in the current trial. If a mixture of Beta distributions with two or more mixture components is required to describe the control response distribution the method of Morita et al.^12^ can be used to determine the *ESS*. The *ESS* approximation obtained here from optimising the parameters of a single Beta distribution were similar to those obtained using the approximate *ESS* formula by Neuenschwander et al.^16^

The original paper for the commensurate prior proposed jointly modelling the historical and current control data. This specification allows feedback from the current control data to the historical control parameter estimate. For one historical study in particular, this will draw the response probabilities in the current and historical controls closer, resulting in a larger estimate of *τ* and greater borrowing from the historical data. Incorporating the historical data as a distributional constant allows the historical data information to feed into the estimate of the current control response probability but does not allow the current control data to feed back into the estimate of the response probability in the historical data.^17^ This model can be fitted in WinBugs using the cut function.^18^ We compare a model incorporating the historical data as a distributional constant to the original commensurate prior model proposed by Hobbs et al.^6^, ^7^ with the priors specified as above.

The commensurate prior is difficult to use in an adaptive design setting since MCMC is required for the analysis and optimisation is required to determine the effective sample size of this historical data, therefore simulations can be computationally expensive. We therefore do not consider this approach for the adaptive design described in Section 2.4.

#### 2.2.4 Robust mixture prior

For one historical study, the robust mixture prior is a two‐component mixture prior, with one component based on the historical data and a weakly‐informative component,^10^

(1) $$\pi \left(p_{c}|x_{h},y_{h},m\right)=\left(1-m\right)\times \text{Beta}\left(p_{c}|x_{h},y_{h}\right)+m\times \text{Beta}\left(p_{c}|1,1\right),$$

where *m* is the prior probability that the new trial control arm differs systematically from the historical study. *m* is user specified and will determine how quickly the historical data are discounted as the difference between the historical and current controls increases.

The posterior distribution for the control parameter is then a mixture of Beta distributions with updated mixture probabilities and parameter values as described in Reference 10.

The effective sample size of the prior data is calculated by determining the effective sample size of the posterior distribution of *p*_*c*_ as described in Reference 10 using the method of Morita et al.^12^ and subtracting the number of current controls randomised. More detail on how the *ESS* is calculated is given in Data S1 for this paper and in the supplementary material of Reference 10.

The robust mixture prior probabilities can be chosen at the design stage to cap the maximum type I error across all possible true current control response probabilities. As with the equivalence probability weight approach, for a design where the historical data are treated as additional information with no trial adaptation, numerical optimisation can be used to determine the initial weights for the robust mixture prior that control the maximum type I error across all possible true control response probabilities at a desired level. For an adaptive design, plotting the maximum type I error distribution for a range of initial robust mixture prior probabilities and using interpolation may be a quicker approach to choosing the probability that controls the maximum type I error at a specified level.

### 2.3 Assessing agreement ‐ Proposed methods

The power prior with a fixed power has the nice property that the power has a meaningful interpretation of the amount of agreement between the current and historical data. For binary data the fixed power prior results in a Beta posterior distribution for which the effective sample size of the historical data is easy to calculate. However, choosing a value for the power is challenging, in the next section we propose two approaches for assessing agreement between the historical and current control data and therefore determining a fixed value for *α*_0_ , which we denote *w*.

The aim of the two methods proposed in this section is to assess the agreement between the historical and current control data and to use this to determine the weight given to the historical data in the final analysis. When outcome data are binary, there is one unknown parameter, the response probability. We compare the distribution of the response probability in the historical and current controls to determine the agreement.

The two methods proposed estimate a weight, anywhere from zero and one, where zero represents no historical data borrowing and one represents pooling of the historical and current control data. The aim of these approaches is to obtain a high weight when there is agreement between the historical and current control data and also to recognise conflict and discount the historical data, obtaining a low weight when there is disagreement between the historical and current controls. The probability weight assesses the disagreement between the current and historical control data using the tail area probabilities, looking at the difference between the current and historical data distributions gives an indication of the conflict between the current and historical data.

#### 2.3.1 Probability weight

Assuming Beta distributions for the historical and current control response probabilities,

*p*_*h*_ ∼ Beta(*x*_*h*_, *y*_*h*_), *p*_*c*_ ∼ Beta(*x*_*c*_, *y*_*c*_).

The probability weight assesses whether current and historical data are compatible based on tail area probabilities and is given by,

$$w=2\times \min\;\left\{\mathrm{Pr}\left(p_{c}>p_{h}\right),1-\mathrm{Pr}\left(p_{c}>p_{h}\right)\right\},$$

as proposed by Thompson,^19^ where,

$$\mathrm{Pr}\left(p_{c}>p_{h}\right)=\int_{0}^{1}\int_{p_{h}}^{1}\frac{p_{h}^{x_{h}-1}{\left(1-p_{h}\right)}^{y_{h}-1}}{B\left(x_{h},y_{h}\right)}\frac{p_{c}^{x_{c}-1}{\left(1-p_{c}\right)}^{y_{c}-1}}{B\left(x_{c},y_{c}\right)}{\mathrm{dp}}_{c}{\mathrm{dp}}_{h},$$

and $B\left(a,b\right)=\frac{\Gamma \left(a\right)\Gamma \left(b\right)}{\Gamma \left(a+b\right)}$.

The probability weight can be calculated exactly and quickly using the iterative procedure described by Cook^20^ when at least one of *x*_*h*_ , *y*_*h*_ , *x*_*c*_ or *y*_*c*_ are integer.

We use the probability weight as a fixed weight for the power prior and therefore for one historical study, the *EHSS* is then the probability multiplied by the historical study sample size *wn*_*h*_ , as illustrated in Section 2.2.1.

One limitation of the probability weight approach is that it has no tuning parameter which allows the user to specify an opinion on what is an acceptable level of agreement between the current and historical control data. A tuning parameter allows some control in the design on how much historical data is incorporated into the analysis for different levels of agreement between the current and historical controls. The equivalence probability weight described in the next section addresses this limitation.

#### 2.3.2 Equivalence probability weight

We describe two equivalence probability weights. The rationale for these weights is to determine how much agreement there is between the historical and current control data based on a user specified range that represents a region of acceptable deviation of the current and historical controls. The one‐sample approach assumes the historical data response probability is the fixed truth and assesses the equivalence of the current controls to the fixed historical response probability. The two‐sample approach acknowledges that the historical data is itself a sample and incorporates this additional uncertainty.

##### One‐sample

Assuming the historical response probability is fixed at the historical sample estimate (${\hat{p}}_{h}$). We choose an equivalence interval around the historical response probability $\left({\hat{p}}_{h}-\delta ,{\hat{p}}_{h}+\delta \right)$, where *δ* is the equivalence bound. The weight is the probability that the current control response probability lies within this interval. We assume a normal approximation to the Beta distribution for the response probability in the current controls. A symmetric equivalence interval around the historical response probability is used. The equivalence probability weight is then given by,

$$w=\mathrm{Pr}\left({\hat{p}}_{h}-\delta <p_{c}<{\hat{p}}_{h}+\delta \right)$$

$$=\Phi \left(\frac{{\hat{p}}_{h}+\delta -{\hat{p}}_{c}}{\sqrt{\frac{{\hat{p}}_{c}\left(1-{\hat{p}}_{c}\right)}{n_{c}}}}\right)-\Phi \left(\frac{{\hat{p}}_{h}-\delta -{\hat{p}}_{c}}{\sqrt{\frac{{\hat{p}}_{c}\left(1-{\hat{p}}_{c}\right)}{n_{c}}}}\right)$$

##### Two‐sample

We assume a normal distribution for both the response probability in the historical data and the current controls and calculate the probability that the difference distribution lies within the chosen equivalence bounds,

$$w=\mathrm{Pr}\left(-\delta <p_{c}-p_{h}<\delta \right)$$

$$=\Phi \left(\frac{\delta -\left({\hat{p}}_{c}-{\hat{p}}_{h}\right)}{\sqrt{\frac{{\hat{p}}_{c}\left(1-{\hat{p}}_{c}\right)}{n_{c}}+\frac{{\hat{p}}_{h}\left(1-{\hat{p}}_{h}\right)}{n_{h}}}}\right)-\Phi \left(\frac{-\delta -\left({\hat{p}}_{c}-{\hat{p}}_{h}\right)}{\sqrt{\frac{{\hat{p}}_{c}\left(1-{\hat{p}}_{c}\right)}{n_{c}}+\frac{{\hat{p}}_{h}\left(1-{\hat{p}}_{h}\right)}{n_{h}}}}\right)$$

We use the equivalence probability weight as a fixed weight for the power prior and therefore for one historical study, the *EHSS* is then the equivalence probability multiplied by the historical study sample size *wn*_*h*_ , as illustrated in Section 2.2.1.

##### Choosing the equivalence bounds

wThe equivalence bounds chosen have a large effect on how much historical data are borrowed and how quickly the historical data are discounted when there is disagreement between the historical and current controls. The equivalence bounds therefore have a large effect on the operating characteristics of the current study. Similar to a test of equivalence of an experimental and control treatment, the equivalence bounds are chosen by the study designer based on prior knowledge. The bounds can be chosen to represent a clinically relevant equivalence distance, which forms a region of acceptable deviation of the current control data from the historical.

In the absence of knowledge about acceptable equivalence bounds, the equivalence bounds can be chosen to minimise trial risk based on statistical properties of the study design. For example controlling the maximum type I error across all possible current control response probabilities. A concern when using historical data is the risk of inflating the type I error. If the design parameters of the current study are chosen to strictly control the type I error when incorporating historical data, there is likely to be little or no benefit in terms of decision‐making power to the historical data design over a standard trial design.^21^ However, the equivalence bounds can be chosen, so that the maximum type I error across all possible true current control probabilities is capped at a chosen value, minimising the risk if the current and historical control probabilities do in fact differ. Alternative approaches are to control the maximum type I error over a plausible range of possible true control responses, rather than over all possible responses, or to control the average type I error, where the expectation is taken over a prior distribution for the current control response probability.

Further considerations in choosing the equivalence bounds are:

- The equivalence bounds should be less than the treatment effect to be detected when comparing the experimental treatment to control in the current trial;
- Narrow equivalence bounds will require large amounts of data to achieve a high weight even when the historical and current controls are in complete agreement. Using the one‐sample equivalence probability weight approach, the weight depends on the current control sample size and using the two‐sample equivalence probability weight approach, the weight depends on both the current control and historical sample sizes. To avoid the historical data having a larger influence on the control parameter estimate than the current control data, one may wish to only consider equivalence bounds that give an *EHSS* less than the current control study sample size at complete agreement between the historical and current controls;
- The equivalence bounds should be chosen to give a larger weight to the historical data around agreement between the current and historical control data and a smaller weight when there is substantial disagreement between the current and historical controls.
- The equivalence bounds can be chosen to govern how quickly the discounting of the historical data occurs as the difference between the historical and current controls increases;
- Criteria other than the maximum type I error could be used for choosing the equivalence bounds, such as controlling the maximum mean square error or the maximum *EHSS* or the average I error given a prior distribution for the current control response probability.

### 2.4 Design and analysis approaches

For the current study comparing the experimental treatment to control our primary analysis of interest is a hypothesis test of *H*_0_ : *p*_*c*_ = *p*_*t*_ against *H*_1_ : *p*_*c*_ < *p*_*t*_ , therefore we are only testing for a positive effect of the experimental treatment.

We use the two‐stage adaptive design proposed by Schmidli et al.,^10^ where the allocation ratio is adapted after the first stage. The number of control and experimental treatment patients randomised in stage 1 ( *n*_*c*1_ and *n*_*t*1_ respectively) and the total number of patients required per treatment group ( *n*_*c*_ and *n*_*t*_ ) are fixed:

Stage 1: Randomise *n*_*t*1_ to the experimental treatment and *n*_*c*1_ to control;

Calculate the effective sample size of the prior data for the control arm, *ESS*_*c*1_ , using the first stage control data, the historical data and the prior assumed for the control response probability before the historical data are observed.

Stage 2: Randomise *n*_*t*_ − *n*_*t*1_ − *ESS*_*t*1_ patients to the treatment arm and the maximum of *n*_*c*_ − *n*_*c*1_ − *ESS*_*c*1_ and *nmin* patients to the control arm (max[ *n*_*c*_ − *n*_*c*1_ − *ESS*_*c*1_ ; *nmin* ]).

Re‐calculate the effective sample size of the prior data for the control group at the end of the study when there is a larger number of current controls available to assess the agreement between the current and historical control data.

*ESS*_*t*1_ is the effective sample size of the prior data for the treatment group and *nmin* is a pre‐specified fixed minimum number of control patients to be randomised in stage 2. For the control group, *ESS*_*c*1_ takes into account the agreement between the historical and current control data. How the *ESS*_*c*1_ is calculated for each historical data method is described in the next section.

The design here replaces some of the current controls with historical controls when the historical and current control data agree. This is to reduce the sample size of the current study and reduce the number of patients that will receive the control treatment in the current study.

Alternatively, a fixed design could be used. The sample size for the current trial is calculated assuming no historical data are available and the historical data are incorporated into the final analysis of the current trial as additional information when there is agreement between the historical and current controls. When there is agreement between the historical and current controls, the additional information results in a study with increased power and precision compared to the no‐borrowing design.

#### 2.4.1 Modified power prior using a summary measure of the marginal distribution of *α*_0_ , probability and equivalence probability weight

We assume an initial vague Beta(1,1) prior on the control response probability before the historical data are observed. This prior is updated with the first stage control data at the interim analysis. At the interim analysis, the weight ( *w*_1_ ) to be given to the historical data is calculated using the modified power prior, probability or equivalence probability weight approach. The weight is calculated as described in Sections 2.2.1, 2.2.2 or 2.3, using the current control data collected up to the interim analysis and the historical data. The *ESS*_*c*1_ ( *w*_1_ *n*_*h*_ + 2) is calculated and the number of control patients to be randomised in stage 2 is determined. At the end of the study the weight is re‐calculated using all of the current study control data ( *w*_2_ ), *w*_2_ is the weight given to the historical data in the final analysis. Similarly, we assume a vague Beta(1,1) prior for the experimental treatment response probability which is updated after stage 2 of the trial, therefore the effective sample size of the prior for the experimental treatment group is 2. The initial vague Beta(1,1) prior distribution on the control response probability and the Beta(1,1) prior distribution on the experimental treatment response probability are assumed to be independent.

The posterior distributions at the end of the study for the control and experimental treatment group are given by,

(2) $$\begin{array}{ll} \pi \left(p_{c}|x_{h},y_{h},x_{c1},x_{c2},y_{c1},y_{c2}\right) & ∼\text{Beta}\left(1+x_{h}w_{2}+x_{c1}+x_{c2},1+y_{h}w_{2}+y_{c1}+y_{c2}\right),\\ \pi \left(p_{t}|x_{t1},x_{t2},y_{t1},y_{t2}\right) & ∼\text{Beta}\left(1+x_{t1}+x_{t2},1+y_{t1}+y_{t2}\right), \end{array}$$

where *x*_*c*1_ and *x*_*c*2_ are the number of responses in the control group in stages 1 and 2 of the trial respectively, *y*_*c*1_ and *y*_*c*2_ the non‐responses and similarly with subscript *t* for the experimental treatment group. *w*_2_ is the weight given to the historical data calculated at the end of the study using the historical data and all of the control data from the current trial.

The primary analysis declares trial success if Pr(*p*_*c*_ < *p*_*t*_| Data ) > 0.975. The Pr(*p*_*c*_ < *p*_*t*_| Data ) can be calculated exactly when one of the Beta parameters of the experimental treatment or control posterior distributions in Equation (2) is an integer using the iterative procedure proposed by Cook.^20^ We know that the number of responses in the control and experimental treatment group follow a binomial distribution. Further, the weight given to the historical data is deterministic given the observed number of control responses. Therefore, considering all possible combinations of first and second stage control responses and all possible experimental treatment responses, we can calculate the operating characteristics of the adaptive study design exactly.

Let *x*_*c*1_ = 0, …, *n*_*c*1_ be the number of first stage control responses and $x_{c2}=0,\ldots ,n_{c2|x_{c1}}$ the number of control responses in stage 2, where $n_{c2|x_{c1}}$ denotes the number of controls required in stage 2. The total number of controls randomised in stage 2 ($n_{c2|x_{c1}}$) is dependent on *x*_*c*1_ , *nmin* and *w*_1_ , where *w*_1_ is the weight given to the historical data at the interim analysis and *w*_1_ is deterministic given the historical data and the first stage current control data. Since *x*_*h*_, *y*_*h*_, *n*_*c*1_ and *nmin* are all fixed by design, the number of second stage controls is denoted $n_{c2|x_{c1}}$, dependent only on the number of observed control responses in stage 1 ( *x*_*c*1_ ). *x*_*t*_ = 0, …, *n*_*t*_ denotes all possible experimental treatment responses. The response distributions are given by,

$$\begin{array}{ll} \mathrm{Pr}\left(X_{c1}=x_{c1}|p_{c},n_{c1}\right) & =\left(\begin{array}{c} n_{c1}\\ x_{c1} \end{array}\right){p_{c}}^{x_{c1}}{\left(1-p_{c}\right)}^{n_{c1}-x_{c1}},\\ \mathrm{Pr}\left(X_{c2}=x_{c2}|p_{c},n_{c2|x_{c1}}\right) & =\left(\begin{array}{c} n_{c2|x_{c1}}\\ x_{c2} \end{array}\right){p_{c}}^{x_{c2}}{\left(1-p_{c}\right)}^{n_{c2|x_{c1}}-x_{c2}},\\ \mathrm{Pr}\left(X_{t}=x_{t}|p_{t},n_{t}\right) & =\left(\begin{array}{c} n_{t}\\ x_{t} \end{array}\right){p_{t}}^{x_{t}}{\left(1-p_{t}\right)}^{n_{t}-x_{t}}. \end{array}$$

The power of the adaptive design is given by,

(3) $$\begin{array}{ll} 1-\beta & =\sum_{x_{c1}=0}^{x_{c1}=n_{c1}}\sum_{x_{c2}=0}^{x_{c2}=n_{c2|x_{c1}}}\sum_{x_{t}=0}^{x_{t}=n_{t}}\mathrm{Pr}\left(X_{c1}=x_{c1}|p_{c},n_{c1}\right)\mathrm{Pr}\left(X_{c2}=x_{c2}|p_{c},n_{c2|x_{c1}}\right)\\ & \times \mathrm{Pr}\left(X_{t}=x_{t}|p_{t},n_{t}\right)1\left(\mathrm{Pr}\left[p_{t}>p_{c}|x_{h},x_{c1},x_{c2},x_{t},n_{c1},n_{c2|x_{c1}},n_{h},n_{t},w_{2}\right]>0.975\right). \end{array}$$

The type I error is calculated using Equation (3) assuming the true underlying experimental treatment response probability *p*_*t*_ is equal to *p*_*c*_ when calculating Pr(*X*_*t*_ = *x*_*t*_| *p*_*t*_, *n*_*t*_).

The expected sample size of the current trial control group ( *ECCSS* ) is given by,

(4) $$\begin{array}{ll} \text{ECCSS} & =n_{c1}+\sum_{x_{c1}=0}^{x_{c1}=n_{c1}}\sum_{x_{c2}=0}^{x_{c2}=n_{c2|x_{c1}}}\mathrm{Pr}\left(X_{c1}=x_{c1}|p_{c},n_{c1}\right)\mathrm{Pr}\left(X_{c2}=x_{c2}|p_{c},n_{c2|x_{c1}}\right)\\ & \times \left(\max\left(n_{c}-n_{c1}-{\mathrm{ESS}}_{c1};\text{nmin}\right)\right), \end{array}$$

and the expected *EHSS* at the end of the current study for a true underlying control response probability is given by,

(5) $$\begin{array}{ll} n_{h}E\left(w_{2}\left(x_{h},n_{h},p_{c},n_{c1},n_{c2|x_{c1}}\right)\right) & =n_{h}\sum_{x_{c1}=0}^{x_{c1}=n_{c1}}\sum_{x_{c2}=0}^{x_{c2}=n_{c2|x_{c1}}}\mathrm{Pr}\left(X_{c1}=x_{c1}|p_{c},n_{c1}\right)\\ & \times \mathrm{Pr}\left(X_{c2}=x_{c2}|p_{c},n_{c2|x_{c1}}\right)w_{2}\left(x_{c1},x_{c2},n_{c1},n_{c2|x_{c1}},x_{h},n_{h}\right). \end{array}$$

The expected total sample size of the control group ( *ECSS* ) including both the current trial controls and the weighted historical data could be greater or less than the total number of controls required *n*_*c*_ . The weight given to the historical data is re‐calculated at the end of the study ( *w*_2_ ), where the level of agreement between the historical and current controls may have changed from the level of agreement at the interim analysis. Further, even when no extra controls are required in stage 2 of the trial, *nmin* controls are randomised to ensure a randomised comparison can be made in both stages of the trial.

The mean squared error is given by,

(6) $$\begin{array}{ll} E{\left({\hat{p}}_{c}-p_{c}\right)}^{2}= & \sum_{x_{c1}=0}^{x_{c1}=n_{c1}}\sum_{x_{c2}=0}^{x_{c2}=n_{c2|x_{c1}}}\mathrm{Pr}\left(X_{c1}=x_{c1}|p_{c},n_{c1}\right)\mathrm{Pr}\left(X_{c2}=x_{c2}|p_{c},n_{c2|x_{c1}}\right)\\ & {\left(\left(\frac{x_{h}w_{2}\left(x_{c1},x_{c2},n_{c1},n_{c2|x_{c1}},x_{h},n_{h}\right)+x_{c1}+x_{c2}}{n_{h}w_{2}\left(x_{c1},x_{c2},n_{c1},n_{c2|x_{c1}},x_{h},n_{h}\right)+n_{c1}+n_{c2|x_{c1}}}\right)-p_{c}\right)}^{2}. \end{array}$$

##### Equivalence bounds that control the maximum type I error

For a design where the weighted historical data are incorporated as additional information at the end of the current study, numerical optimisation can be used to determine the equivalence bounds that control the maximum type I error at a chosen value by minimising the function,

$${\left(\max_{p_{c}\in \left[0,1\right]}\left(\hat{\alpha }|\delta \right)-\alpha^{*}\right)}^{2}$$

where $\max_{p_{c}\in \left[0,1\right]}\left(\hat{\alpha }|\delta \right)$ is the maximum type I error across all true control proportions in the current study for a given equivalence bound. *α* ^*^ is the required maximum type I error.

Optimisation is quick for the additional information design because there are few possible combinations of control and experimental treatment responses. For the two‐stage adaptive design the number of possible combinations of first and second stage control responses and experimental treatment responses is much higher. The weight given to the historical data also has to be calculated at the interim analysis and re‐calculated at the final analysis. The adaptive design optimisation did not converge for the example used in this paper. Depending on the number of patients in each treatment group, an approximation of the equivalence bounds that control the maximum type I error can be determined by plotting the maximum error distribution for a range of equivalence bounds and using interpolation to choose the bounds that control the maximum type I error across all possible true control response probabilities.

#### 2.4.2 Robust mixture prior analysis approach

The prior distribution for the control arm is given in Equation (1). A Beta(1,1) prior is assumed for the experimental treatment response probability. The posterior distribution at the end of the study for the control parameter is then a mixture of Beta distributions with updated mixture probabilities and parameter values as described in Reference 10. The mixture probabilities and parameter values are updated at the interim analysis and again at the final analysis.

The probability that treatment response is greater than control using the robust mixture prior approach for the adaptive design can also be calculated exactly using Cook's method.^20^ Cook's method^20^ is applied comparing the experimental treatment response probability distribution to each mixture component of the control posterior distribution separately. These probabilities are weighted by the posterior mixture probability to obtain the overall Pr(*p*_*t*_ > *p*_*c*_| Data ).

The power, type I error, *EHSS*, *ECCSS* and mean squared error are then calculated using Equations (3), (4), (5), (6). The robust mixture prior requires the Morita algorithm to calculate the *ESS* of the posterior distribution for the control response probability. More detail on how the *ESS* is calculated is given in Data S1 for this paper and in supplementary material of Reference 10. To calculate the power, type I error, *ECCSS* and mean squared error for the adaptive design, only the *ESS* at the interim analysis is required. To calculate the *ECSS* at the end of the study, the *ESS* of the posterior distribution at the end of the study is required and this will be computationally expensive using the Morita algorithm for the control group given all of the first and second stage response combinations for the adaptive design. Furthermore, it has been suggested that the Morita method gives optimistic estimates of the *EHSS*.^22^ A simpler alternative approximation of the *EHSS* proposed by Neuenschwander et al.^16^ could be used but gives different results to the Morita algorithm.^22^

#### 2.4.3 No historical data design

All of the historical data methods above are compared to a design not incorporating any historical data. The operating characteristics for this design are calculated using the analysis approach described in Section 2.4.1 assuming the weight is zero.

## 3 RESULTS

To illustrate and compare the methods described in Section 2, we extend the example from Viele et al.^2^ to be an adaptive design.

We wish to design a standard two‐arm randomised trial which requires 200 patients in both the experimental treatment and control arm with a one‐sided type I error rate of 2.5%. There are 100 ( *n*_*h*_ ) historical control patients available with a response probability of 65%. Assuming 65% is also the true response probability in the current controls, a standard design would have approximately 76% power to detect a difference between the experimental treatment and control response probabilities of 12%.

Section 3.1 compares the weight or prior *ESS* obtained from assessing the agreement between the historical and current controls for a given observed number of control responses using each of the methods described in Section 2. In Section 3.1 we assume there are 100 historical and 100 current control patients. Section 3.2 then explores the operating characteristics of the adaptive design proposed in Section 2 using the modified power prior taking a summary measure of the marginal distribution of *α*_0_ , robust mixture prior, probability weight and equivalence probability weight approaches.

### 3.1 Fixed weights

**FIGURE 1:**
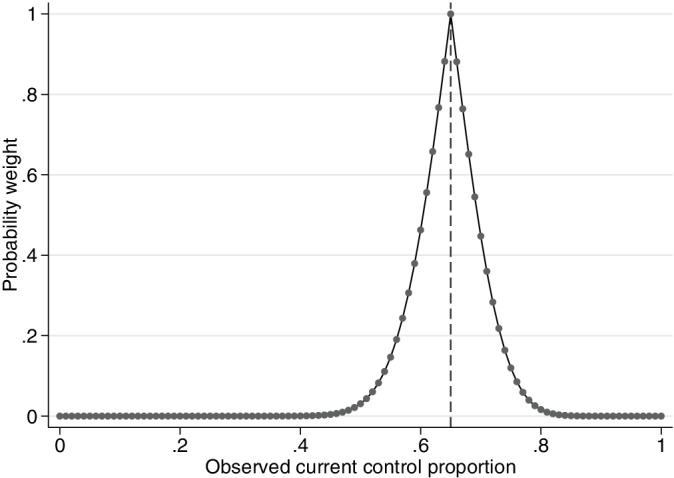
Probability weight for different observed current control response probabilities. Example, historical data 65/100 responses, 100 current controls. The vertical dashed line represents complete agreement between the historical and current control proportions

Figure 1 illustrates the probability weight obtained for different observed control response probabilities in the current trial.

Using the probability weight approach, when the observed historical and current control data completely agree, the historical data are given a high weight in the final analysis. The weight decreases quickly to zero as the difference in the observed response probabilities increases. At a 15% difference in the observed current control and historical response probabilities, the probability weight obtained is close to zero. The disadvantage of the probability weight approach is that the rate at which the historical data are discounted is fixed.

**FIGURE 2:**
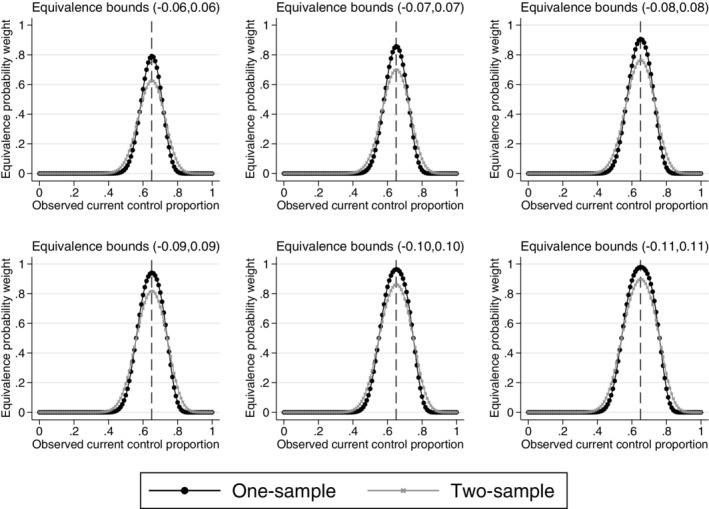
One and two‐sample equivalence probability weights for different observed current control response probabilities and different equivalence bounds. Example, historical data 65/100 responses, 100 current controls. The vertical dashed lines represent complete agreement between the historical and current control proportions

Figure 2 shows the equivalence probability weights for different equivalence bounds and different observed current control response probabilities for both the one and two‐sample methods.

Narrow equivalence bounds require a large amount of data to yield a high weight at complete observed agreement between the current and historical controls. Wider equivalence bounds give a higher weight to the historical data, both when the observed historical and current control data agrees and disagrees. With all equivalence bounds, the weight decreases to zero as the difference in the observed response probabilities increases. The choice of equivalence bounds can therefore determine the amount of borrowing and the rate at which the historical data are discounted. Due to the increased variability in the two‐sample equivalence probability weight from the historical data uncertainty, the two‐sample approach gives a lower weight when there is complete agreement between the observed current and historical control response probabilities compared to the one‐sample equivalence probability weight. The two‐sample equivalence probability weight decreases to zero at a slower rate than the one‐sample equivalence probability weight as the difference between the historical and current control data increases.

**FIGURE 3:**
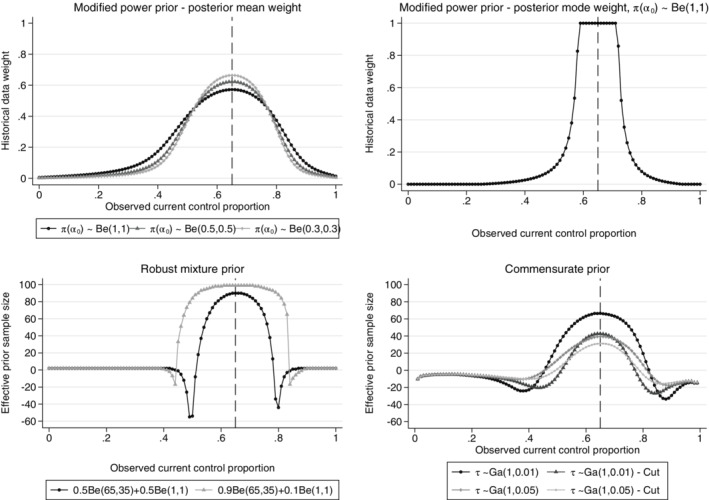
Posterior mean of *α*_0_ for different priors on *α*_0_ (top left), posterior mode of *α*_0_ assuming a Beta(1,1) prior on *α*_0_ (top right), prior effective sample size of the robust mixture prior assuming different initial weights on the informative component of the mixture prior (bottom left), prior effective sample size of the commensurate prior with different priors on *τ* using the cut function and without the cut function (bottom right), the weights and prior *ESS* are given for different observed current control response probabilities. Example, historical data 65/100 responses, 100 current controls

Figure 3 shows the weights or the prior *ESS* obtained using historical data methods proposed in the literature. The robust mixture prior with 90% probability on the historical data component of the mixture prior gives a high *ESS*, even when there is substantial disagreement (15% difference) between the historical and observed current controls. The robust mixture prior with 50% probability on the historical data component discounts the historical data quickly as the difference increases. The robust mixture prior and commensurate prior approaches give a negative prior *ESS* for a range of differences between the observed historical and current data, as illustrated in Figure 3. This occurs when the posterior distribution has a larger variance than either the prior or the likelihood when there is moderate conflict between the current and historical control data. For the modified power prior and commensurate prior, the priors on the power and commensurability parameter are difficult to choose and the priors explored in this paper do not induce a larger amount of historical data borrowing when there is agreement between the observed historical and current controls and both discount slowly as the difference between the historical and current controls increases. Using the mean of the marginal distribution of *α*_0_ as a fixed weight to down‐weight the historical data gives a weight close to 0.5 when the historical and current observed response probabilities are the same and discounts slowly to zero as the difference increases, even when the sample sizes are large.^5^ The quasi‐dichotomous priors give a higher weight at agreement and discount more quickly to zero than the Beta(1,1) prior. Using the mode of the marginal distribution of the power as a fixed weight, assuming a initial Beta(1,1) prior on the power gives a weight of one to the historical data (pooling of the historical and current controls) for a range of response probabilities around complete agreement of the observed data and discounts quickly to zero as the difference between the observed current and historical control response probabilities increases. However, for a Beta(1,1) prior, at complete agreement in the observed historical and current control response probabilities, the posterior distribution of *α*_0_ does not change much from the prior distribution for *α*_0_ , since the information value from two observed response rates is small.

### 3.2 Simulation study of the frequentist operating characteristics for the adaptive design

We explore the operating characteristics of the adaptive design described in Section 2.4 for a range of true control response probabilities in the current study. The experimental treatment response probability is always assumed to be 12% higher than the control response probability. There are 100 ( *n*_*h*_ ) historical control patients available with a response probability of 65%. The interim analysis is conducted after 100 patients have been randomised to both the control and experimental treatment arm ( *n*_*c*1_ = *n*_*t*1_ = 100), and *nmin* = 20. The total sample size required per treatment group is *n*_*c*_ = *n*_*t*_ = 200. A Beta(1,1) prior is assumed for the control response probability before the historical data are observed. A Beta(1,1) prior is also assumed for the experimental treatment response probability for which there are no historical data available.

**FIGURE 4:**
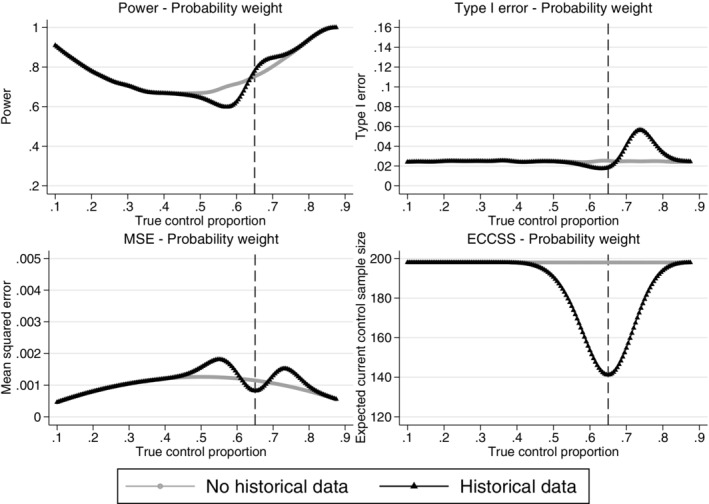
Comparison of the power, type I error, mean squared error and expected current control sample size across different true current control proportions for the adaptive design using the probability weight approach and a standard design incorporating no historical data. Example, historical data 65/100 responses, *n*_*c*_ = *n*_*t*_ = 200, *n*_*c*1_ = 100, *nmin* = 20 and Δ = 12%. The vertical dashed lines represent complete agreement between the historical and current control proportions. ECCSS, expected current control sample size; MSE, mean squared error

**FIGURE 5:**
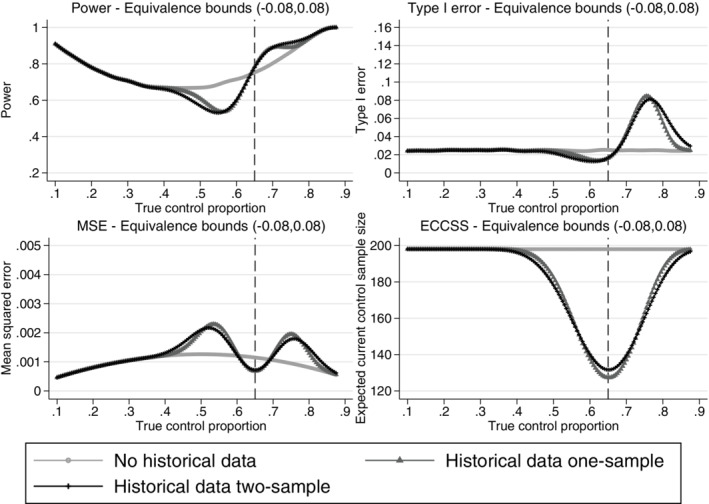
Comparison of the power, type I error, mean squared error and expected current control sample size across different true current control proportions for the adaptive design using the one‐sample and two‐sample equivalence probability weight approaches with 8% equivalence bounds and a standard design incorporating no historical data. Example, historical data 65/100 responses, *n*_*c*_ = *n*_*t*_ = 200, *n*_*c*1_ = 100, *nmin* = 20 and Δ = 12%. The vertical dashed lines represent complete agreement between the historical and current control proportions. ECCSS, expected current control sample size; MSE, mean squared error

**FIGURE 6:**
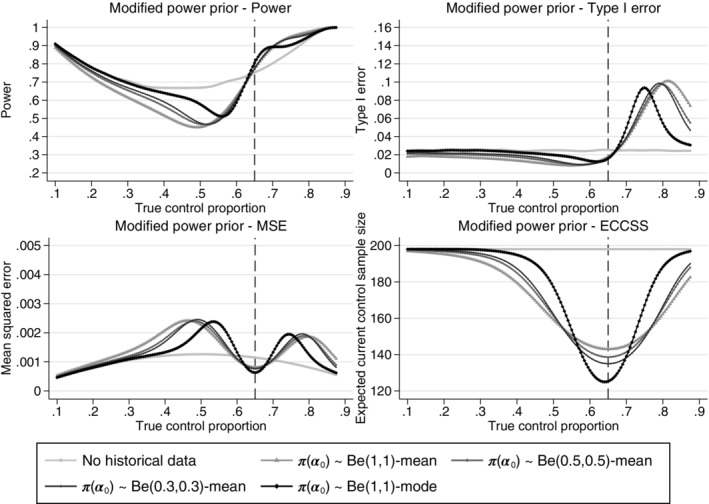
Comparison of the power, type I error, mean squared error and expected current control sample size across different true current control proportions for the adaptive design using the power prior with a summary measure of the marginal distribution of *α*_0_ as a fixed weight, assuming different priors on the power and a standard design incorporating no historical data. Example, historical data 65/100 responses, *n*_*c*_ = *n*_*t*_ = 200, *n*_*c*1_ = 100, *nmin* = 20 and Δ = 12%. The vertical dashed lines represent complete agreement between the historical and current control proportions. ECCSS, expected current control sample size; MSE, mean squared error

**FIGURE 7:**
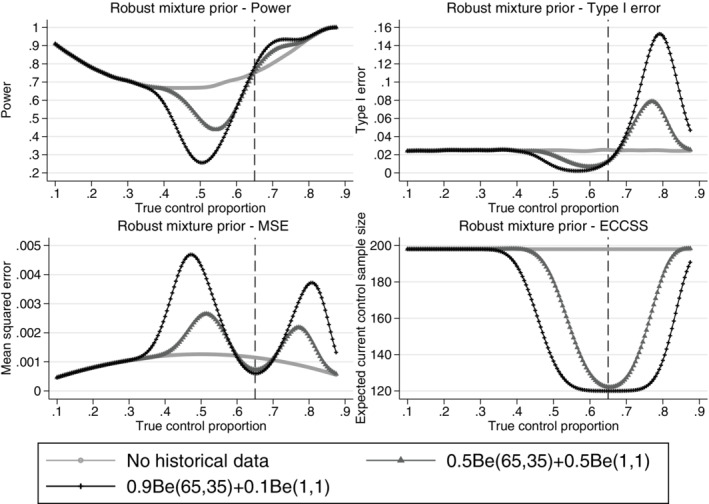
Comparison of the power, type I error, mean squared error and expected current control sample size across different true current control proportions for the adaptive design using the robust mixture prior approach with 0.9 and 0.5 initial weight on the informative component of the mixture prior and a standard design incorporating no historical data. Example, historical data 65/100 responses, *n*_*c*_ = *n*_*t*_ = 200, *n*_*c*1_ = 100, *nmin* = 20 and Δ = 12%. The vertical dashed lines represent complete agreement between the historical and current control proportions. ECCSS, expected current control sample size; MSE, mean squared error

From Figures 4, 5, 6, 7, the operating characteristics of all methods depend on how quickly the historical data are discounted and the direction of the difference between the historical and current control response probabilities. All methods perform similarly when the true control proportion is close to 0.65 (the historical response probability). The power is slightly increased compared to a design not incorporating the historical data and the type I error is lower than the desired 2.5% level. This is because at least 20 control patients are randomised in stage 2 of the trial even if all of the historical controls are used and therefore no current controls are required in stage 2 to make the control sample size 200. When the current control response probability is higher than the historical, the historical data draws the estimated control response probability down, increasing the treatment effect estimate and inflating the type I error. When the current control response probability is less than the historical, the estimated treatment effect is reduced and the power is reduced when compared to a design not incorporating any historical data. When the difference between the historical and current controls is large, the historical data is given zero weight in the analysis and the operating characteristics revert back to those of a standard trial design. As shown for the equivalence probability weight in Data S1, the expected current control sample size varies slightly around 200 (approximately between 195 and 200) therefore the sample size will slightly affect the operating characteristics, however most of the impact on the operating characteristics comes from the difference between the historical and current controls. The main advantage of the adaptive design is the reduction in the number of control patients required in the current study when the historical and current control data are in agreement. All methods result in a substantial reduction in the number of controls required in the current study when the historical and current control data are in agreement. However, as shown by the mean squared error there is a sweet spot around agreement between the historical and current controls where the historical data approaches are beneficial and there are ranges of disagreement where the historical data approaches perform worse compared to a standard trial design borrowing no historical data.

Note that for the adaptive design the expected total sample size of the control group (current trial controls + weighted historical controls) could be greater or less than the required number of controls of 200. The prior *ESS* is re‐calculated at the end of the study, where the level of agreement between the historical and current controls may have changed from the agreement at the interim analysis. We do not calculate the expected total control sample size for the robust mixture prior approach since this would require using the Morita algorithm to calculate the *ESS* of the posterior distribution at the end of the study for all possible combinations of first and second stage responses for all possible second stage sample sizes. Instead we provide a summary of the expected current trial control sample size, which does not require calculating the *EHSS* at the end of the study. The moments matching approach to estimating the *EHSS* could be used for the robust mixture prior approach which is fast to calculate.^16^

#### 3.2.1 Probability and equivalence probability weight

Figure 4 shows the probability weight quickly reverts back to a standard design (giving the historical data zero weight) as the difference between the historical and current controls increases and the maximum possible type I error is 5.6%. The probability weight is fixed and therefore does not allow control over the rate at which the historical data are discounted.

Figure 5 shows the design characteristics of the one‐sample and two‐sample equivalence probability weight approaches with 8% equivalence bounds. 8% equivalence bounds were chosen here as sensible bounds for a 12% treatment effect to illustrate the equivalence method. However, as discussed in Section 2.3.2, the equivalence bounds would be chosen based on prior knowledge or to control the operating characteristics of the study design at a desired level. Narrower equivalence bounds would borrow less historical data both when the historical and current controls agree and disagree. This would reduce the maximum possible type I error but would also increase the *ECCSS* at complete agreement between the current and historical control data. The choice of equivalence bounds allows control over the maximum inflation in type I error, this is explored further in Section 2.4.1. Note that for both the probability and equivalence probability weight approaches there is a range of true control proportions around agreement with the historical data response proportion where incorporating the historical data improves the design characteristics (illustrated by the lower MSE compared to a design using no historical data), however, where there is a difference between the true current control proportion and the historical proportion, but the difference is not large enough for the historical data to be completely discounted, the designs can have negative effects (illustrated by the higher MSE compared to a design using no historical data). The advantage of these adaptive designs is in the reduction of the current control sample size when there is agreement between the historical and current control data, approximately 60 patients are saved compared to a standard trial design using the probability weight and approximately 70 patients for the equivalence probability weight approaches with 8% equivalence bounds. If the historical and current control data are in agreement the type I error is controlled at the 2.5% level. The worst inflation in type I error for this example of the equivalence approach with 8% equivalence bounds is a type I error of approximately 8%. The expected total control sample size for the equivalence probability weight with different equivalence bounds is illustrated in Data S1. The expected equivalence probability weight at the interim and final analysis of the adaptive design for different equivalence bounds and the design characteristics for the equivalence probability weight with different equivalence bounds are also shown in Data S1.

#### 3.2.2 Methods proposed in the literature

Figure 6 shows the operating characteristics of the proposed adaptive design for the modified power prior using the mean or the mode of the posterior distribution of *α*_0_ as a fixed weight. The modified power prior using the mean as a fixed weight discounts the historical data slowly as the difference between the current and historical controls increases and a large difference is required before the historical data are completely discounted and the operating characteristics of a standard trial design are achieved. The maximum type I error is similar for all priors considered here on the power. The parameters of the prior on *α*_0_ cannot be easily chosen to control the maximum type I error or the rate of discounting of the historical data. Using the mode of the marginal distribution of the power results in good operating characteristics. However, there is no flexibility to control the maximum type I error across all possible true control proportions in the current study.

Figure 7 shows the operating characteristics for the robust mixture prior approach. The robust mixture prior allows control of the rate at which the historical data are discounted through the choice of the initial robust mixture prior probabilities. The analysis is conjugate and therefore quick for calculating the power and type I error of the adaptive design. When the prior weight on the informative component of the mixture distribution is 0.9, the robust mixture prior borrows a lot of historical data, reducing the number of current controls required in stage 2 to the compulsory 20 patients. However, with the large initial weight on the historical data mixture component, there is a large range of true control proportions where the mean squared error is higher than for a standard trial design and the maximum type I error is approximately 0.16, much higher than the standard trial design level of 0.025. Reducing the initial weight on the historical data component of the mixture prior results in quicker discounting of the historical data as conflict increases and results in a smaller maximum type I error and a smaller range of current control proportions where the mean squared error is higher than the no historical data design.

As in Section 3.2.1 for the modified power prior and robust mixture prior there is a small range of true control proportions around agreement with the historical data where the historical data methods improve the design characteristics (lower MSE compared to no historical data design), however outside this range and before the difference between the historical and current control gets large enough for the historical data to be completely discounted, there is a range of true control proportions where the historical data approaches can result in a larger MSE than the design incorporating no historical data.

#### 3.2.3 Comparison of methods

**FIGURE 8:**
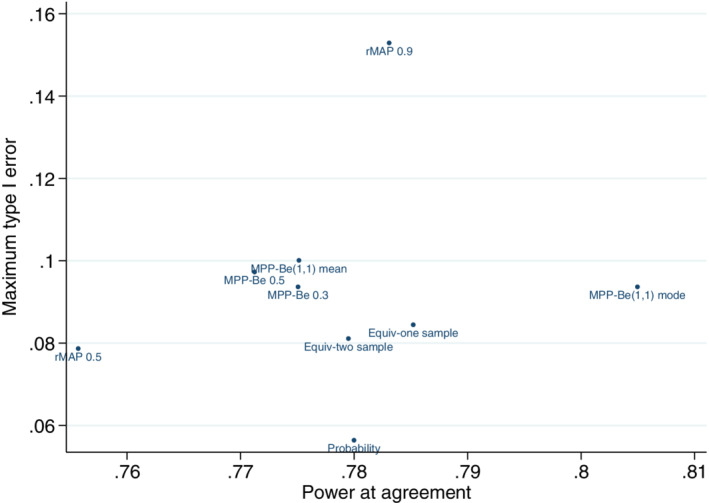
Comparison of maximum type I error and power at complete agreement between the current and historical control parameters for all historical data approaches. Example, historical data 65/100 responses, *n*_*c*_ = *n*_*t*_ = 200, *n*_*c*1_ = 100, *nmin* = 20 and Δ = 12%. Probability, probability weight. Equiv‐one sample, One sample equivalence probability weight with 8% equivalence bounds. Equiv‐two sample, Two sample equivalence probability weight with 8% equivalence bounds. MPP‐Be(1,1)‐mean, modified power prior‐ posterior mean weight ‐ *π*(*α*_0_) ∼ Be(1, 1). MPP‐Be(0.5,0.5)‐mean, modified power prior‐ posterior mean weight ‐ *π*(*α*_0_) ∼ Be(0.5,0.5). MPP‐Be(0.3,0.3)‐mean, modified power prior‐ posterior mean weight ‐ *π*(*α*_0_) ∼ Be(0.3,0.3). MPP‐Be(1,1)‐mode, modified power prior‐ posterior mode weight ‐ *π*(*α*_0_) ∼ Be(1, 1). rMAP 0.9, robust mixture prior ‐ 0.9Be(65,35) + 0.1Be(1,1). rMAP 0.5, robust mixture prior ‐ 0.5Be(65,35) + 0.5Be(1,1)

Figure 8 shows a comparison of all of the historical data approaches for the adaptive design by plotting the maximum type I error for each approach against the power attained at complete agreement between the current and historical control parameters.

Points in the lower right quadrant show the best performing approaches where the maximum type I error is used as the metric of interest. The lower right quadrant indicate methods that have the highest power when the historical and current control parameters are in agreement but also give the lowest maximum type I error when the historical and current control parameters differ. Alternative methods for defining the type I error can be used and are described in the discussion section. The best performing method may differ when using different definitions for the type I error. Similarly, if the user specified values are varied such as the bounds in the equivalence weight approach or the initial mixture prior weight, the best performing method may differ. The ideal situation would be to have a single measure combining the type I error and power into a single value to allow the methods to be ordered, this approach could be considered for future research.

#### 3.2.4 Equivalence bounds that control the maximum type I error

**FIGURE 9:**
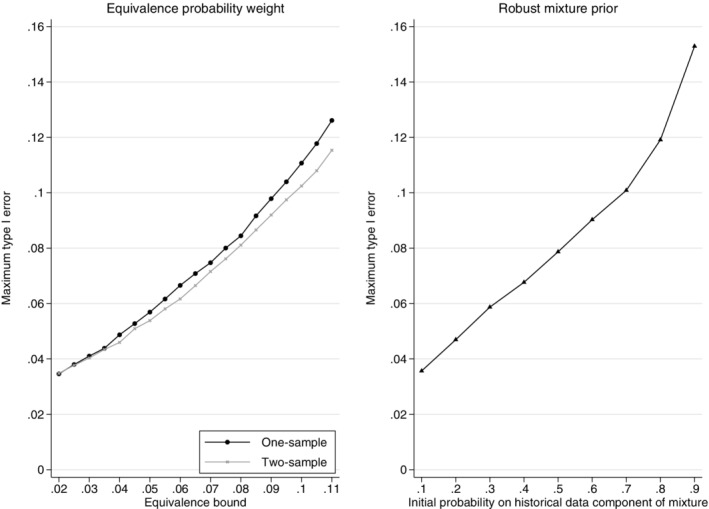
Distribution of the maximum possible type I error across a range of equivalence bounds using the one‐sample and two‐sample equivalence probability weight approaches (left plot) and the distribution of the maximum possible type I error across a range of initial mixture prior probabilities on the informative component of the robust mixture prior for the adaptive design (right plot). Example, historical data 65/100 responses, *n*_*c*_ = *n*_*t*_ = 200, *n*_*c*1_ = 100, *nmin* = 20 and Δ = 12%

Figure 9 illustrates the maximum type I error across all true control proportions in the current study for different equivalence bounds. Both the one‐sample and two‐sample equivalence probability weight approaches are shown. Note that controlling the maximum possible type I error at 2.5% (the type I error for a design not incorporating historical data) is only possible when incorporating no historical data. Controlling the maximum possible type I error across all true current control proportions to be 2.5% while incorporating historical data would require a larger sample size than a standard trial design. The equivalence bounds that control the maximum type I error at approximately 5% for the one‐sample equivalence probability weight approach are ±0.042 and for the two‐sample equivalence probability weight approach are approximately ±0.044. It is often more efficient to determine the equivalence bounds that control the maximum type I error across the current control proportions by plotting the distribution of the maximum type I error for a range of current control proportions and using interpolation to determine the bounds that give the desired maximum possible type I error, rather than using optimisation. Determining the maximum type I error for the robust mixture prior approach for different initial mixture prior probabilities requires calculating the effective sample size at the interim analysis for all possible responses for each initial mixture prior probability using the Morita algorithm.

## 4 DISCUSSION

In this paper, two intuitive and computationally tractable approaches for assessing agreement between historical and current control data are proposed. An adaptive design that replaces current controls with historical controls is then utilised which allows the possibility of borrowing historical data when there is agreement with the current control data. We propose using the method described by Cook^20^ to calculate Pr(*p*_*t*_ > *p*_*c*_) which allows the operating characteristics of the proposed design to be calculated exactly and quickly. The equivalence probability weight approach is flexible and allows control over how quickly the historical data are discounted when there is disagreement between the current and historical control data. The maximum inflation in type I error across all possible current control response probabilities can be calculated and controlled by the choice of equivalence bounds. Further, the equivalence probability weight is an intuitive way to think about discounting historical data and should provide an approach that is easy to discuss with clinicians about utilising historical data and the effect that using historical data has on the design characteristics of the current study. Using an adaptive design gives the advantage of reducing the trial sample size or duration when the historical and current controls agree but safeguards the trial by allowing more current controls to be randomised in the second stage of the trial when there is disagreement between the current and historical controls at the interim. This adaptive approach could potentially be beneficial in making early investment decisions during the trial if the historical data agree with the current data and therefore a larger amount of data is available to be used in decision making.

We do not advocate the use of historical data in trials that can easily recruit the required number of patients under a standard design. But where recruitment is slow, the patient population is small or for making early within company decisions for a trial (e.g., using historical data in a phase II trial analysis to potentially start phase III planning) historical data methods could potentially increase the power, reduce the duration, or allow early decisions to be made for a trial which would not be feasible under a standard design. Furthermore, controlling the maximum type I error is a strict constraint on using historical data methods. Given that the historical study is carefully selected, the different between the historical and current control data that gives the maximum type I error may be unrealistic and a fairer approach maybe to look at the maximum type I error across a range of true control proportions that are likely in the current study, or an average type I error across a plausible range of true control proportions.

All historical data methods work in a similar way, discounting the historical data when there is disagreement, but the methods differ in the amount of information borrowed and the rate of discounting as conflict increases. In this example, we found the modified power prior with a range of priors on the power discounts the historical data slowly, giving some weight to the historical data in the analysis even when there is a large difference between the current and historical controls. The commensurate prior was difficult to use in an adaptive design setting since for a single simulation two MCMC models and an optimisation to calculate the *ESS* at the interim is required which is computationally expensive. The priors on the parameters that govern borrowing of the historical data in both the modified power prior and commensurate prior approach have a large effect on the weighting of the historical data and require careful thought. The robust mixture prior approach can give a weight close to one when there is agreement between the current and historical control data, depending on the initial weights chosen and the initial weights have a meaningful interpretation of the prior opinion of the historical and current control data agreement. It is important to carefully select the initial weight given to the historical data component in the mixture prior since this will affect how much historical data is borrowed for different levels of current and historical control agreement. Calculating the *ESS* for the robust mixture prior approach using the Morita method was slow and negative values can be obtained which requires setting the *ESS* to zero when used in an adaptive design setting. Otherwise the historical data design would require more controls to be randomised than a standard trial design not incorporating any historical data. The Morita method has been suggested to give optimistic *ESS* values and alternative approaches, such as matching moments, may be more appropriate and faster to calculate.^22^ For one historical study, the effect of the mixture probability on the historical data component of the robust mixture prior needs exploration but can be used to control the maximum type I error across a range of true control response probabilities in the current trial. The robust mixture prior and equivalence probability weight approaches were the preferred methods here due to the meaningful interpretation of the tuning parameters (the initial mixture prior weights and the equivalence bounds), both approaches allow control of the operating characteristics easily through the tuning parameters and the analyses are straightforward. Furthermore, both of these approaches can be tuned to how much information they borrow at agreement and how quickly the historical data is discounted as the difference increases between the current and historical control data.

In this paper, following the example in Reference 2 which provided an initial comparison of some historical data methods, we assume that the true control response probability is a fixed value. It could be assumed that the current control response probability has a distribution, which would be used for sampling in the simulations, furthermore the type I error could be averaged over the assumed distribution for the current control response probability. The operating characteristics are presented as a function of the true control proportion in the current study. The expected total control sample size varies slightly around 200, however the impact of the operating characteristics is mostly from the difference between the current and historical controls.

The probability and equivalence approaches were chosen to give a high weight to the historical data when there is complete agreement between the historical and current controls. A setting where we may not want to give the historical data a high weight is when there is a substantial amount of historical control data and we do not want to overwhelm the data from the current trial. In this case a maximum weight may be imposed.

An assumption made throughout this paper is that the current trial information is the most reliable information and where differences are seen between the current and historical data, the historical data are discounted in the final analysis. It was not considered that the current control group may by chance not be representative of the population response probability for the control treatment.

For all historical data methods, careful thought is required as to what is appropriate historical data. Control data may be available from multiple studies spanning many years. If the historical studies selected are very heterogeneous, the prior *ESS* may be low and there may be no benefit to a historical data borrowing trial design.^9^ In this paper we have focused on the specific problem of having only one relevant historical study, where it is not possible to obtain an accurate estimate of the between study heterogeneity and therefore some prior judgement of the agreement between the current and historical control data is required for all of the methods explored. Methods for incorporating multiple historical studies have been proposed for the robust MAP prior, commensurate prior and power prior methods.^3^, ^4^, ^6^, ^7^, ^8^, ^10^ The probability and equivalence weight methods could be extended to multiple historical studies by first combining the historical studies and assessing the agreement between the combined historical studies and the current trial control data or alternatively by assessing the agreement between each historical study and the current control group and down‐weighting each historical study individually. Future work will explore the probability and equivalence probability weight approaches when there are multiple historical studies. The best approach may differ depending on the number of historical studies. For a small number of historical studies, where estimating the between study heterogeneity is difficult, incorporating the studies individually may be the best approach. For many historical studies a meta‐analysis approach may be best.

The examples considered here were consistent with a confirmatory trial design in terms of the operating characteristics chosen and therefore the required sample size. We did not explore how the methods worked when the current study was an early phase trial design, which typically have a smaller sample size and allow a higher type I error rate. Further, we did not consider a setting where there is a large amount of historical data compared to the sample size of the current control arm. Therefore, further work would look at the effect of different sample sizes in the historical and current trial and trials and different design characteristics. The adaptive design considered in this paper only incorporated one interim analysis. As data on the current control arm accrues, the accuracy of the estimate of the agreement between the historical and current controls will also improve. Possible future work could look at the optimal time to perform the interim analyses and the optimal number of interim analyses that are required to improve the efficiency of the design but still allow the design to be computationally and logistically plausible. The probability and equivalence probability weight methods have been extended to normally distributed outcome data.^23^

In the recent literature, alternative approaches have been proposed for determining a fixed power when using the power prior method. An empirical Bayes approach has been proposed by Gravestock and Held.^15^ For binary data, this approach is the same as using the mode of the marginal posterior distribution of the power as a fixed weight when assuming a flat Beta(1,1) prior on the power, which is illustrated throughout this paper. Ibrahim et al.^24^ propose various methods to facilitate the choice of a fixed power for a normal linear model, these are: a penalised likelihood type criterion; marginal likelihood criterion; deviance information criterion; and pseudo‐marginal likelihood criterion. Finally, Pan et al.^25^ propose the calibrated power prior where the power is defined as a function of a congruence measure between the historical and current data. The calibrated power prior was only illustrated for normally distributed outcome data with known variance. Future work would compare these approaches to the equivalence probability weight approach proposed in this paper.

## CONFLICT OF INTEREST

The author declares that there is no conflict of interest that could be perceived as prejudicing the impartiality of the research reported.

## Supporting information


**Data S1.** Supporting Information.

## Data Availability

All data used in this paper were randomly generated. The code used to generate the data is available from the corresponding author upon reasonable request.
