# Supplementary material for: A novel equivalence probability weighted power prior for using historical control data in an adaptive clinical trial design: A comparison to standard methods
Source: Pharm Stat. 2021 Jan 20;20(3):462–84. doi: 10.1002/pst.2088 (PMC8611797; doi:10.1002/pst.2088)
Supplement: Supplementary file 1 — Data S1. Supporting Information. [file PST-20-462-s001.pdf]

1 Supplementary information

1.1 Calculating the effective sample size for the robust mixture prior

The agreement between the historical and current controls is summarised using the ESS. The ESS is determined by calculating the ESS of the control posterior distribution incorporating both the historical and randomised controls in the current trial and subtracting the number of randomised controls [1]. The ESS of the posterior distribution is calculated using the method of Morita et al. [2], which is outlined below.

For binary data, the Morita algorithm searches for a distribution with known sample size that has the same information at the mean as the posterior distribution for the current control response probability distribution. The sample size of the posterior distribution is then estimated to be the sample size of the created distribution. Here we use the algorithm by Morita but the information is compared at the mode of the distribution as proposed by Schmidli et al. [1]. The original paper by Morita et al. [2] shows that when comparing the information at the mean, the ESS matches the commonly used ESS value for the Beta distribution of the sum of the parameter values [2]. However, the mode is used here since this seems a more appropriate measure when the posterior mixture distribution may be bimodal.

The control response probability posterior distribution is a mixture of Beta distributions and the mode is found by searching over the function values to find the maximum. The observed information at the mode is then approximated using a quadratic approximation. Let $\tilde{p}_c$ be the mode of the current control posterior distribution. The observed information is defined as,

$$I = -\left.\frac{d^2 log\pi(p_c)}{dp_c^2}\right|_{p_c=\tilde{p}_c}$$

The second derivative is calculated using a quadratic approximation. This is the quadratic function whose first and second derivatives are the same as those of the distribution of p_c at the mode. The formula is given by,

$$\pi(p_c) \approx \pi(\tilde{p}_c) + \dot{\pi}(\tilde{p}_c)(p_c - \tilde{p}_c) + \frac{1}{2}\ddot{\pi}(\tilde{p}_c)(p_c - \tilde{p}_c)^2.$$

This quadratic approximation is the second-order Taylor polynomial for the posterior mixture distribution of p_c at $p_c = \tilde{p}_c$.

The observed information of the control response probability posterior distribution is compared to a posterior distribution constructed from a non-informative prior and has a known sample size. The expected information for the posterior distribution with sample size m under a weakly-informative prior is,

$$\mathbf{E}(I_0(m)) = -\sum_{x_c=0}^{x_c=m}\left\{\left.\frac{d^2 log(\pi_0(p_c \mid x_c))}{dp_c^2}\right|_{p_c=\tilde{p}_c}\right\}\pi(x_c).$$

where $\pi_0(p_c \mid x_c)$ is a Beta distribution with sample size m and $\pi(x_c)$ is the prior predictive distribution with respect to the informative prior $\pi(p_c)$, which is a mixture of Beta-Binomial distributions. As an initial non-informative prior we choose a

Beta($\tilde{p}_c/c, (1-\tilde{p}_c)/c$), where c is a large constant, here it is chosen to be 100. We then loop over all sample sizes m up to a reasonable maximum, which is chosen to be the sum of the Beta component of the mixture distribution with the largest parameter values plus 10. The ESS is the largest m such that $\mathbf{E}(I_0(m)) < I$ [1].

1.2 Expected total control sample size for the one-sample equivalence probability weight with varying equivalence bounds

Section 3 of the main paper explores the design characteristics for an example using the adaptive design proposed in Section 2.4 of the main paper. Figure 1 illustrates the $ECSS$ across a range of true current control proportions for the adaptive design example using the one-sample equivalence probability weight approach with varying equivalence bounds.

Figure 1: $ECSS$ across different true current control proportions for the adaptive design using the one-sample equivalence probability weight approach with 4%, 6%, 8% and 10% equivalence bounds and a standard design incorporating no historical data. Example, historical data 65/100 responses, $n_c = n_t = 200$, $n_{c1} = 100$, $nmin = 20$ and $\Delta = 12\%$. The vertical dashed lines represents complete agreement between the historical and current control proportions.

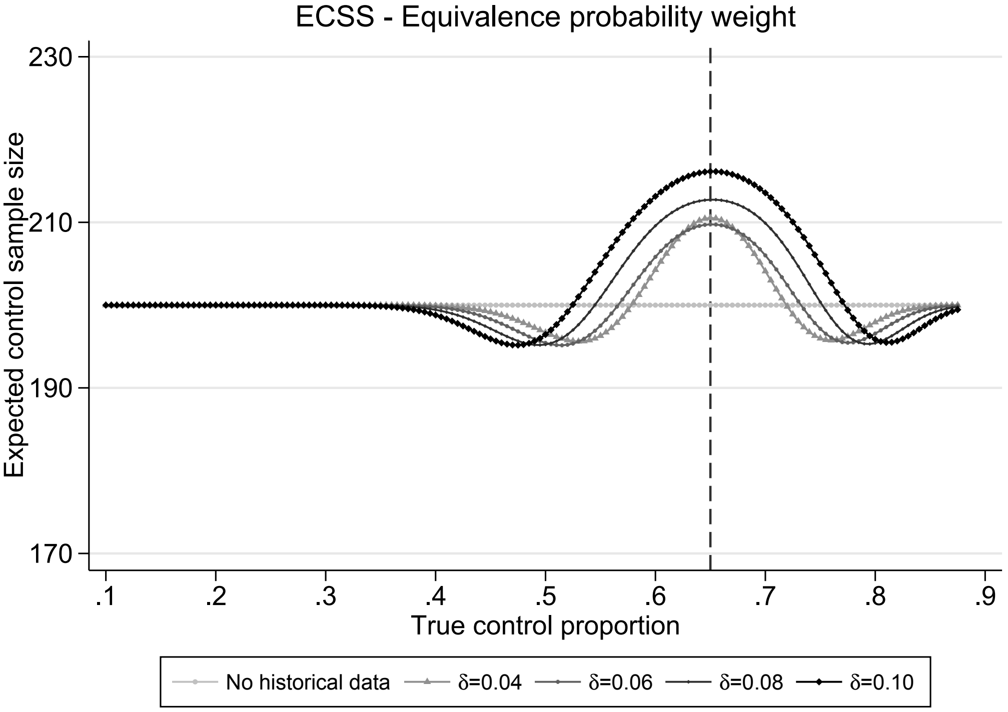

For a range of current control response probabilities around complete agreement between the current and historical data, the $ECSS$ for the adaptive design is slightly above the 200 patients required under a standard trial design. This increase in sample size is due to the minimum requirement of 20 controls to be randomised in stage 2 of the trial and also due to the weight given to the historical data changing from the interim analysis to the final analysis. The $ECSS$ is also slightly below 200 for a range of current control

proportions due to the change in the weight calculated at the interim analysis and the final analysis. The change in the weight from the interim to the final analysis is illustrated in Section 1.3.

1.3 Expected equivalence probability weights

For the adaptive design proposed in Section 3 of the main paper, the equivalence probability weight is calculated at the interim analysis and this weight is used to choose how many current controls to randomise in stage 2 of the trial. The weight given to the historical data is re-calculated at the end of the trial and this weight is used to discount the historical data in the final analysis.

Figure 2 illustrates the difference in the expected weights at the interim analysis and the final analysis for the example considered throughout this paper for the equivalence probability weight.

Figure 2: Expected probability weight at the interim analysis and at the end of the study across different true current control proportions for the adaptive design using the one-sample and two-sample equivalence probability weight approaches with 4%, 6%, 8% and 10% equivalence bounds and a standard design incorporating no historical data. Example, historical data 65/100 responses, $n_c = n_t = 200$, $n_{c1} = 100$, $nmin = 20$ and $\Delta = 12\%$. The vertical dashed lines represents complete agreement between the historical and current control proportions.

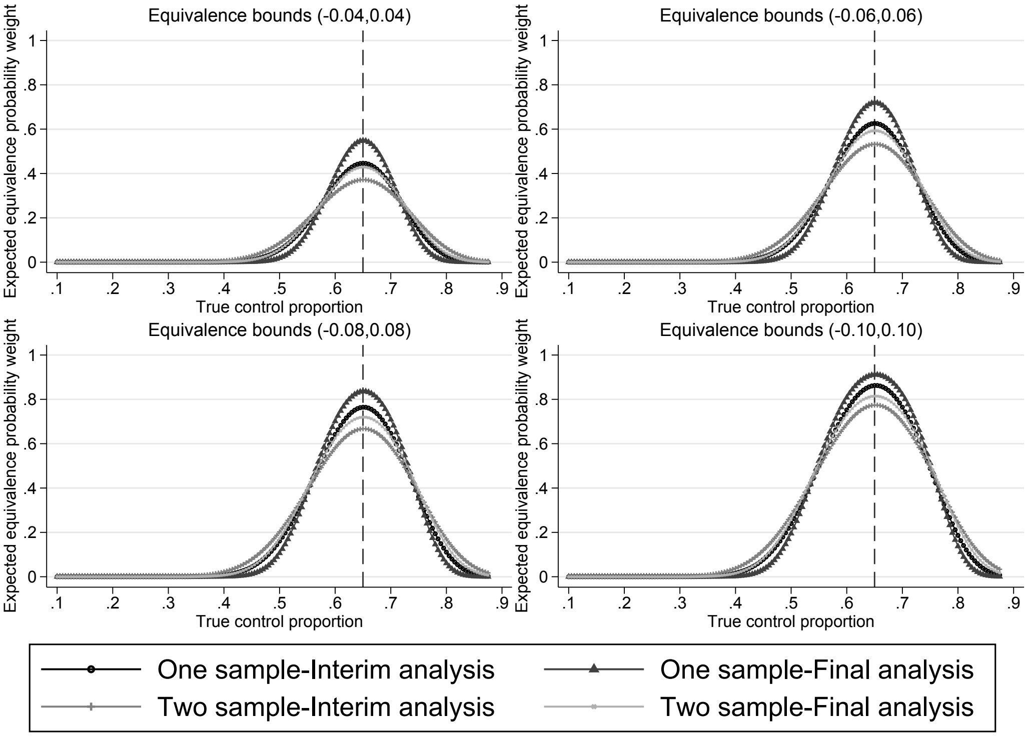

At the end of the study, when the sample size of the current controls is generally larger than at the interim analysis, on average the weight is slightly higher at complete agreement and discounts to zero at a quicker rate than at the interim analysis. The difference

between the equivalence probability weight at the interim and final analysis differs depending on the equivalence bounds chosen.

1.4 Operating characteristics for the one-sample equivalence probability weight approach with varying equivalence bounds

Figure 3 illustrates the operating characteristics for the one-sample equivalence probability weight approach for different equivalence bounds (4%,6%,8% and 10%).

Figure 3: Comparison of the power, type I error, mean squared error and expected current control sample size across different true current control proportions for the adaptive design using the one-sample equivalence probability weight approaches with 4%, 6%, 8% and 10% equivalence bounds and a standard design incorporating no historical data. Viele example, historical data 65/100 responses, $n_c = n_t = 200$, $n_{c1} = 100$, $nmin = 20$ and $\Delta = 12\%$. The vertical dashed lines represent complete agreement between the historical and current control proportions.

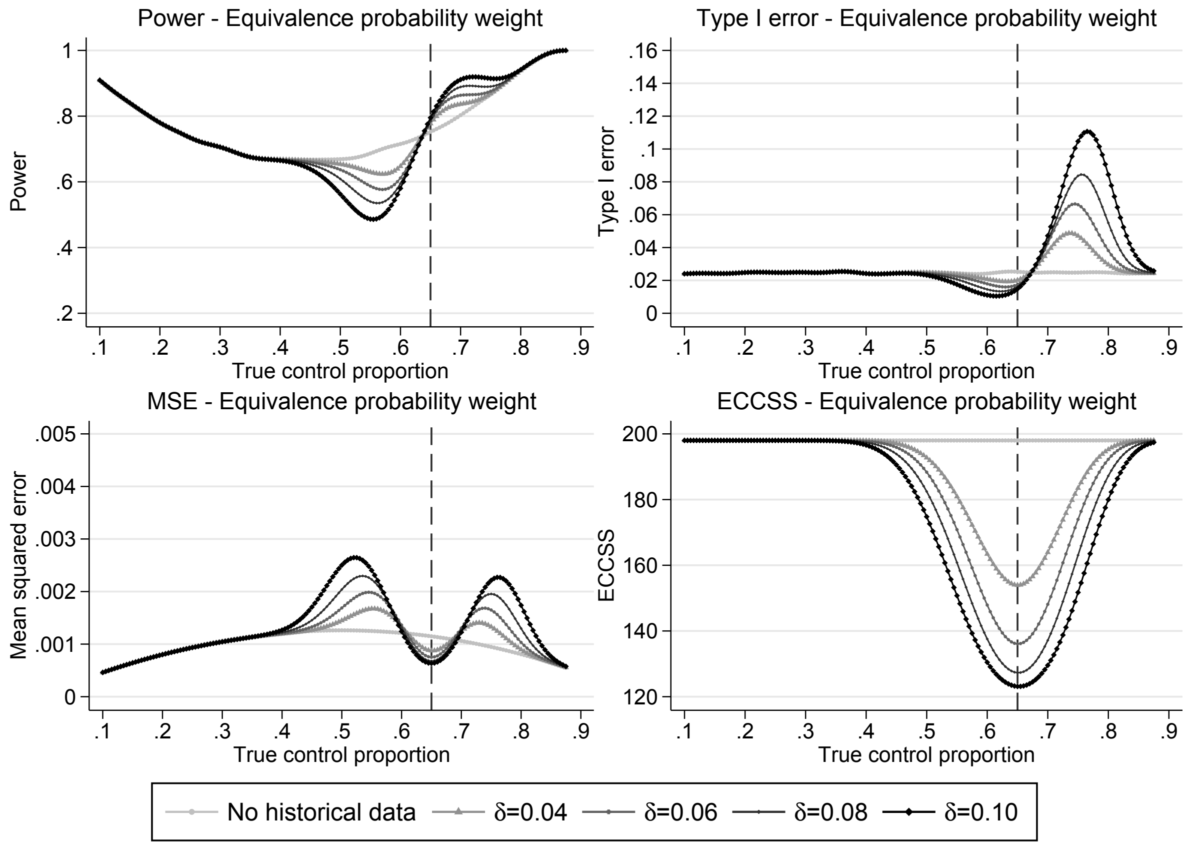

MSE - Mean squared error, ECCSS - Expected current control sample size.

As the equivalence bounds increase, at complete agreement between the historical and current control data, the amount of historical data borrowed and the power both increase, however this is at the expense of a higher maximum possible type I error rate for larger

equivalence bounds when the estimated current control response probability is larger than the historical response probability.

References

[1] Heinz Schmidli, Sandro Gsteiger, Satrajit Roychoudhury, Anthony O'Hagan, David Spiegelhalter, and Beat Neuenschwander. Robust meta-analytic-predictive priors in clinical trials with historical control information. *Biometrics*, 70(4):1023–1032, 2014.

[2] Satoshi Morita, Peter F Thall, and Peter Müller. Determining the effective sample size of a parametric prior. *Biometrics*, 64(2):595–602, 2008.
